# An Immunity-Associated lncRNA Signature for Predicting Prognosis in Gastric Adenocarcinoma

**DOI:** 10.1155/2022/3035073

**Published:** 2022-04-25

**Authors:** Xiaowen Zhao, Pingfan Wu, Dongling Liu, Changtian Li, Ling Xue, Zhe Liu, Meng Zhu, Jie Yang, Ziyi Chen, Yaling Li, Yali She

**Affiliations:** ^1^Department of Pathology, School of Basic Medicine, Gansu University of Chinese Medicine, Lanzhou, Gansu, China; ^2^Laboratory of Preclinical Medicine of the 940th Hospital of Joint Logistics Support Force of Chinese People's Liberation Army, Key Laboratory of Stem Cells and Gene Drug of Gansu Province, Lanzhou, Gansu, China; ^3^Provincial-Level Key Laboratory of Molecular Medicine of Major Diseases and Study on Prevention and Treatment of Traditional Chinese Medicine, Gansu University of Chinese Medicine, Lanzhou, Gansu, China

## Abstract

**Background:**

Gastric adenocarcinoma (GAD) is one of the most common tumors in the world and the prognosis is still very poor.

**Objective:**

We sought to identify reliable prognostic biomarkers for the progression of GAD and the sensitivity to drug therapy.

**Method:**

The RNA sequencing data of GAD was downloaded from the Cancer Genome Atlas (TCGA) database and used for analysis. Differentially expressed, immune-related lncRNA (DEIRlncRNA) was characterized by differential analysis and correlation analysis. Univariate Cox regression analysis was used to identify DEIRlncRNA associated with prognosis. Least absolute shrinkage and selection operator (LASSO) regression analysis allowed us to determine a signature composed of eight IRlncRNAs. Based on this signature, we further performed gene set enrichment analysis (GSEA) and somatic mutation analysis to evaluate the ability of this signature to predict prognosis.

**Results:**

In total, 72 immune-related lncRNAs (DEIRlncRNAs) with prognostic value were identified. These lncRNAs were used to construct a model containing eight immune-related lncRNAs (8-IRlncRNAs). Based on this risk model, we divided GAD patients into high-risk and low-risk groups. The analysis showed that the prognosis of the two groups was different and that the high-risk group had worse overall survival (OS). Immune cell infiltration analysis showed that the proportion of memory B cells increased in the high-risk group while the proportion of macrophages M1, T cells, CD4 memory-activated cells, and T cell follicular helpers decreased. GSEA results showed that 8-IRlncRNA was significantly enriched in tumorigenesis pathways such as myc. The results of somatic mutation analysis showed that the CDH1 gene was significantly mutated in the high-risk group.

**Conclusion:**

A prognostic signature of 8-IRlncRNAs in GAD was established and this signature was able to predict the prognosis of GAD patients.

## 1. Introduction

Gastric cancer (GC) has been identified as the fifth most common cancer and the third most common cause of death in the world [1]. Due to the low rate of early diagnosis of GC, most patients are in an advanced stage or have metastases when they are discovered, and the 5-year survival rate of GC patients is still less than 10% [2]. Gastric adenocarcinoma (GAD) is the most common histological type of GC accounting for >95% [3, 4]. Although there has been improvement in treatment methods, such as chemotherapy, surgery, and targeted therapy, the prognosis of GC is still poor [5, 6]. Reliable biomarkers for progression and sensitivity to drug therapy are greatly needed in GAD.

LncRNA are noncoding RNAs with a length greater than 200 nucleotides and have been shown to be involved in the progression of GC. LncRNA PVT1 promotes GC tumor growth and metastasis by stabilizing FOXM1 protein [7]. LncRNA SNHG11 promotes cell proliferation, differentiation, migration, and invasion in GC and has been shown to be related to the poor prognosis of patients [8]. LncRNAs are key regulators of gene expression in the immune system [9]. At the same time, many reports indicate that lncRNA plays a role in the regulation of the immune system [10]. LncRNA CamK-A participates in the remodeling of the tumor microenvironment by activating Ca^2+^-triggered signal transduction [11]. The progression of cancer, including GC, is related to immune infiltration. Some studies have shown that immune cells, especially tumor-infiltrating lymphocytes, help determine the progression of GC. For example, the CD155/TIGIT signaling pathway inhibits the metabolism of CD8^+^ T cells and promotes the progression of GC [14]. The high density of CD3^+^, CD8^+^, and CD45RO^+^ cells is closely related to the survival of GC patients and regional lymph node metastasis [15]. In addition, the application of immune checkpoint inhibitors (ICIs) improves the survival rate of GC patients in the advanced stage [16, 17]. Programmed death protein 1 (PD-1) inhibitors, including Nivolumab and Pembrolizumab, have been approved for clinical use to improve the 1-year and 2-year survival rates of patients with gastric cancer [18, 19]. However, only a small percentage of patients benefit from immunotherapy [20]. Studies have shown that patients with high levels of microsatellite instability significantly benefited from pembrolizumab, revealing the usefulness of molecular typing and biomarkers in identifying patients who may benefit from immunotherapy inhibitors [21]. Although immune-related long noncoding RNAs (lncRNAs) have been identified as potential biomarkers, there is still a lack of immune-related lncRNA signatures that can predict the response of GC immunotherapy.

Based on this, our study analyzed the RNA sequencing data of GAD obtained from TCGA and immune-related mRNA obtained from the Molecular Signatures Database. We established an 8-immune-related lncRNA (8-IRlncRNA) signature by LASSO Cox regression analysis. GSEA was used to explore immune-related response pathways. Furthermore, we evaluated the predictive potential of this signature from the perspective of immune cell infiltration and ICIs.

## 2. Materials and Methods

### 2.1. Data Sources

The RNA sequencing and clinical information data of GAD was downloaded from the TCGA database (https://portal.gdc.cancer.gov/). The inclusion criteria for the samples in this study were as follows: (1) samples included complete clinicopathological information; (2) samples with an OS time of less than 100 days were excluded. Using these criteria we selected a total of 279 patients including 24 normal samples and 279 cancer samples. The clinicopathological characteristics of the included patients are summarized in Table 1. We extracted 327 immune-related mRNAs (IRmRNAs) from the Molecular Signatures Database (https://www.broadinstitute.org/gsea/msigdb/index.jsp: Immune System Processes M13664, Immune Responses M19817, v4.0).

### 2.2. Differentially Expressed Analysis and Enrichment Analysis

We used the “limma” package in R software (v4.0.4) to perform differentially expressed analysis on GAD samples and normal gastric samples to identify differentially expressed mRNA (DEIRmRNA) and differentially expressed lncRNA (DElncRNA). The selection criteria for DEIRmRNA and DElncRNA were a *P* value <0.05 and fold change criterion |logFC| > 1. In addition, in order to reveal the potential function of DEIRmRNA, the “clusterProfiler” R package was used to perform Gene Ontology (GO) and Kyoto Encyclopedia of Genes and Genomes (KEGG) pathway enrichment analysis. GO analysis included biological process (BP), cell composition (CC), and molecular function (MF). The cutoff criterion was *P* value <0.05.

### 2.3. Identification of Differentially Expressed Immune-Related lncRNA

In order to further identify differentially expressed immune-related lncRNA (DEIRlncRNA), we carried out Pearson correlation analysis between DElncRNA and DEIRmRNA. Pearson correlation coefficient |*R*| > 0.4 and *P* value <0.05 were set as cutoff criteria.

### 2.4. Identification of an Immune-Related Prognostic Signature Based on DEIRlncRNA

We used the “survival” R package to perform univariate Cox regression analysis on the selected DEIRlncRNA to further select the IRlncRNA related to the prognosis of GAD patients. Second, we used LASSO regression analysis to identify a meaningful prognostic signature. As mentioned earlier, a total of 279 GAD samples were used to construct IRlncRNA signature. We randomly divided the 279 GAD samples into training set (*n* = 195) and test set (*n* = 84). The training set was used to explore and construct an IRlncRNA signature. The test set and entire GAD patient cohort (*n* = 279) were used to verify the IRlncRNA signature. Finally, we used these IRlncRNAs in multivariate Cox regression to obtain coefficients and determine 8 IRlncRNAs (8-IRlncRNA) that were significantly related to prognosis. Thereby, a predictive model weighted by their coefficients was established. We constructed a risk score formula for OS to assign a risk score to each patient:

(1) $$\begin{array}{c} \mathrm{risk}\;\mathrm{score}=\sum_{i=1}^{n}\mathrm{Coe}f_{i}*x_{i}, \end{array}$$

where Coef_*i*_ means the coefficients and *x*_*i*_ is the counts value of each lncRNA. Risk scores were computed for all patients included in our study. The low-risk group and the high-risk group were determined by the best cutoff value. Best cutoff value referred to the risk score with the largest difference in survival between the two groups at the lowest log rank *P* value.

### 2.5. The Potential of the 8-IRlncRNA Signature to Predict Prognosis

We used the “timeROC” R package to perform the time-varying receiver operating characteristic (ROC) curve analysis in the training set and draw a Kaplan–Meier (KM) survival curve to compare the OS difference between the high-risk group and the low-risk group for evaluating the predictive value of the 8-IRlncRNA signature. Similarly, the stability and reliability of the 8-IRlncRNA signature were verified through the above analysis in the test set (*n* = 84) and the entire GAD cohort (*n* = 279). The low-risk group and high-risk group were determined by the best cutoff value. A *P* value <0.05 was considered statistically significant.

### 2.6. Correlation Analysis of Immune Cell Infiltration Based on the 8-IRlncRNA Prognostic Signature

CIBERSORT is a computerized method for calculating differences in cell subpopulation composition that can be used to identify predictive and prognostic cellular biomarkers as well as novel therapeutic targets [22]. In order to compare the different immune infiltration levels between high-risk and low-risk groups based on the 8-IRlncRNA prognostic signature, the “CIBERSORT” algorithm was employed to quantify the 22 tumor-infiltrating immune cell proportions in high-risk and low-risk groups.

### 2.7. Gene Set Enrichment Analysis of the 8-IRlncRNA Prognostic Signature

Gene set enrichment analysis (GSEA) is a method used to determine if a predefined gene set can show significant consistent differences in two biological states. Therefore, we performed a GSEA to explore the potential function of the 8-IRlncRNA prognostic signature. The low-risk group and the high-risk group were determined according to the best cutoff value and GSEA software (downloaded from https://software.broadinstitute.org/gsea/index.jsp, v4.1.0) was used in the analysis. The predefined gene set of “hallmark.v6.2.symbols.gmt” was downloaded from the molecular marker database. The false discovery rate (FDR) < 0.25 was defined as the critical value and sorted by normalized enrichment score (NES) value.

### 2.8. Somatic Mutation Analysis

Gene mutation data were downloaded from the TCGA database. Somatic variants were analyzed using the R package “maftools” to compare the overall mutational status of high- and low-risk groups. Significantly mutated genes were also compared between the two risk groups.

## 3. Results

**Figure 1:**
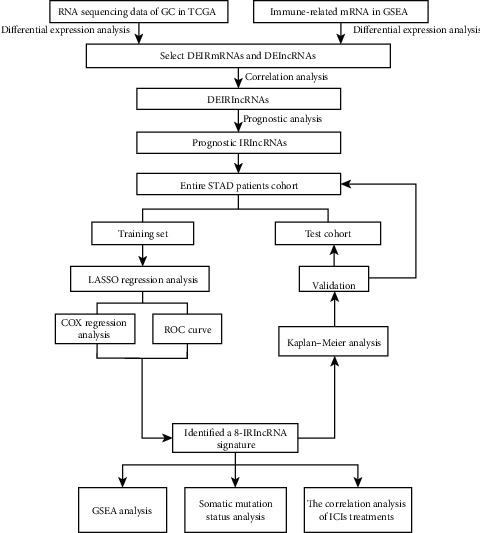
Flow chart showing the analysis process.

Figure 1 is the technical roadmap of this study.

### 3.1. Identification of DElncRNA and DEIRmRNA

Through differential expression analysis of the RNA sequencing data of GAD, we identified a total of 1843 DElncRNAs between GAD and normal stomach samples, including 904 upregulated and 939 downregulated DElncRNAs. We identified 72 DEIRmRNAs by differential expression analysis based on the acquired 327 IRmRNAs. These DElncRNAs and DEIRmRNAs were used for subsequent analysis. In addition, we performed GO and KEGG enrichment analysis to identify functions and mechanisms associated with these DEIRmRNAs. The results of the enrichment analysis are shown in Supplementary Figure 1. These DEIRmRNAs are mainly enriched in immunoglobulin complex formation, G protein-coupled receptor binding, and interleukin 17 (IL-17) signaling pathways.

### 3.2. Construction and Verification of an 8-IRlncRNA Prognostic Signature in the Training Set

**Figure 2:**
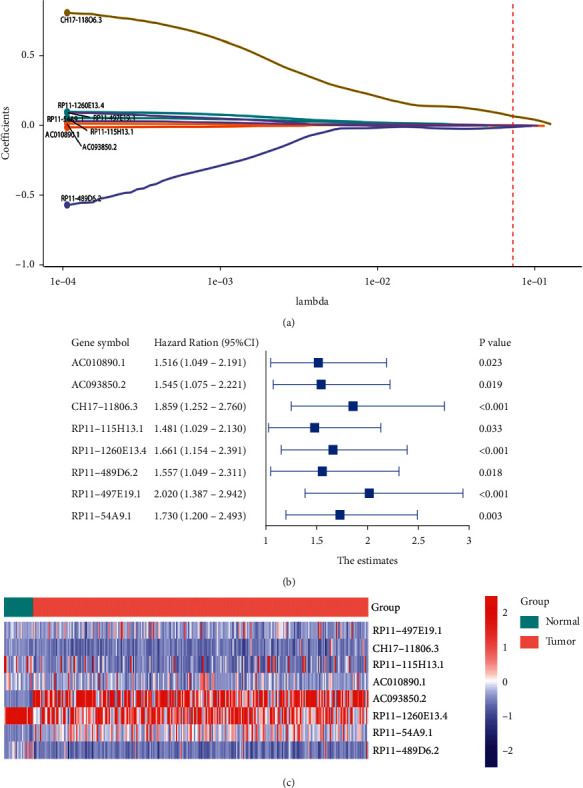
Model construction by choosing prognosis IRlncRNA. (a) LASSO regression analysis identified 8 IRlncRNAs. (b) Forest plot showing the HR and 95% CI of 8 IRlncRNAs through Cox regression analysis. (c) A heatmap of 8 IRlncRNAs in normal and tumor tissues.

After we conducted Pearson correlation analysis on DElncRNAs and DEIRmRNAs, 648 differentially expressed, immune-related lncRNAs (DEIRlncRNAs) were identified. We performed univariate Cox regression analysis on these 648 DEIRlncRNAs for the purpose of selecting IRlncRNAs related to prognosis in GAD patients. A total of 94 prognostically related IRlncRNAs were identified. Then, we used LASSO (Figure 2(a)) and multivariate Cox regression analysis to identify 8 IRlncRNAs which are closely related to the prognosis of GAD in the training set. Detailed information on the 8 IRlncRNAs is shown in Table 2. A forest diagram shows the hazard ratio (HR) and 95% confidence interval (95% CI) of 8 IRlncRNAs (Figure 2(b)). The heatmap of 8 IRlncRNAs in normal and tumor tissues is shown in Figure 2(c). The results show that these 8 IRlncRNAs were all harmful IRlncRNAs. Their univariate Cox HRs were all greater than 1, meaning that patients with high expression of 8 IRlncRNAs may have a poor OS.

**Figure 3:**
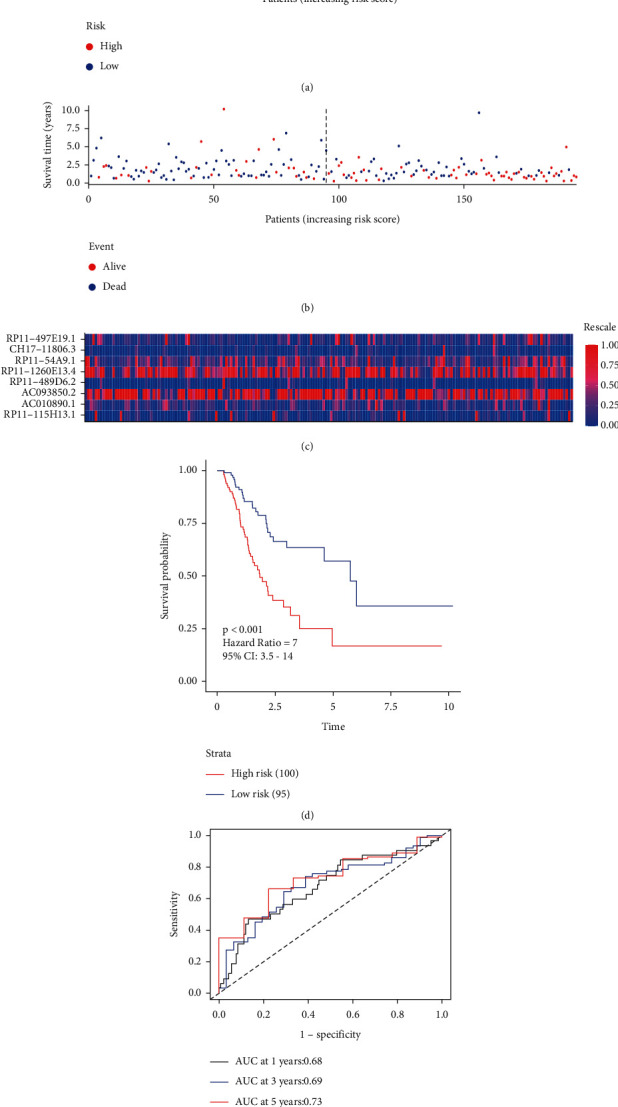
Construction of the prognostic signature based on 8-IRlncRNA in the training set. (a) Patients were divided into high- and low-risk groups based on 8-IRlncRNA in the training set. (b) The survival status of GC patients in the training set. (c) Heatmap of expression profiles of 8-IRlncRNA. (d) Survival analysis of high- and low-risk groups. (e) Time-dependent ROC curve of the 8-IRlncRNA prognostic signature.

To evaluate the prognostic ability of the 8-IRlncRNA signature in GAD and assign a risk score to each patient, we determined the risk score of the 8-IRlncRNA signature. This risk score is based on the expression of these 8 IRlncRNAs and their ability to predict OS. The specific formula is: Risk score = 0.0105512600 × (RP11-497E19.1) + 0.0147859892 × (CH17-118O6.3) + 0.0004966951 × (RP11-54A9.1) + 0.0035751428 × (RP11-1260E13.4) − 0.0008728424 × (RP11-489D6.2) + 0.0014697293 × (AC093850.2) + 0.0081959700 × (AC010890.1) + 0.0003624435 × (RP11-115H13.1). According to the best cutoff value, patients were divided into high-risk groups and low-risk groups (Figure 3(a)). Seven of these eight IRlncRNAs were associated with high-risk (AC010890.1, AC093850.2, CH17-118O6.3, RP11-115H13.1, RP11-1260E13.4, RP11-497E19.1, and RP11-54A9.1, coefficient >0) and one was protective (RP11-489D6.2, coefficient <0).

In the training set, the mortality rate of the high-risk group was higher than that of the low-risk group (Figure 3(b)). The expression levels of the eight lncRNAs in each sample is shown in Figure 3(c). The results of survival analysis (Figure 3(d)) show that the OS of patients in the high-risk group is shorter than that of patients in the low-risk group in the training set. The ROC curve was used to predict the area under the curve (AUC) value for 1-year, 3-year, and 5-year OS. The AUC values were 0.68, 0.69, and 0.73, respectively (Figure 3(e)). These results indicate that the 8-IRlncRNA prognostic signature has excellent sensitivity and specificity.

### 3.3. Verification of the 8-IRlncRNA Prognostic Signature

We also used the above formula to calculate the risk score of the 8-lncRNA signature to verify the predictive ability on OS. Then, the signature was verified in the test set and the entire GAD cohort (Supplementary Figure 2; Supplementary Figure 3). The verification results are consistent with the findings in the training set. Based on the above analysis results, the 8-IRlncRNA signature is considered to be a good prognostic label for OS.

### 3.4. The Correlation between Clinicopathologic Characteristics, Molecular Subtype, and the 8-IRlncRNA Signature in GAD

**Figure 4:**
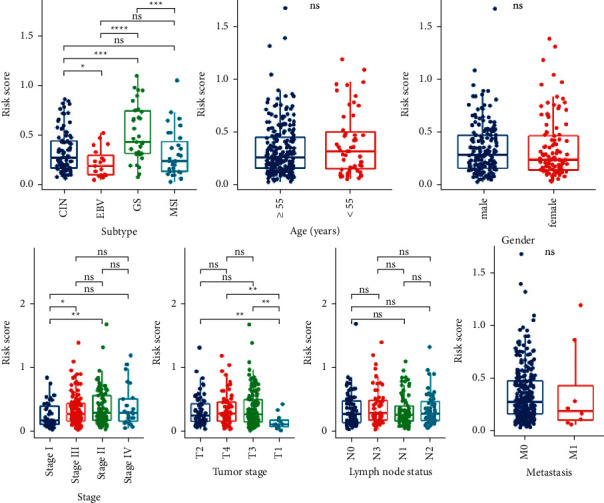
Correlation analysis between risk scores and molecular subtypes, clinicopathological characteristics. (a) The molecular subtype analysis. (b)–(g) The clinicopathological characteristics analysis. ^∗^*P* < 0.05. ^∗∗^*P* < 0.01. ^∗∗∗^*P* < 0.001.

To further validate the prognostic value and explore the applicability of the immune-related signature, we attempted to determine if clinicopathological features and molecular subtype are associated with the risk score. A total of 279 GAD patients (96 patients in the high-risk group and 183 patients in the low-risk group) were included in this part of the analysis. Among the molecular subtypes, the genomically stable (GS) subtype had the highest risk score, while the Epstein–Barr virus (EBV) subtype had the lowest risk score. Meanwhile, the risk scores of chromosomal instability (CIN) and microsatellite instability (MSI) subtypes were between GS and EBV (Figure 4(a)). The analysis of clinicopathological characteristics showed that the risk score is related to tumor stage, but not related to age, gender, pathological grade, or lymph node status (Figures 4(b)–4(g)).

### 3.5. Pathway Enrichment Analysis

**Figure 5:**
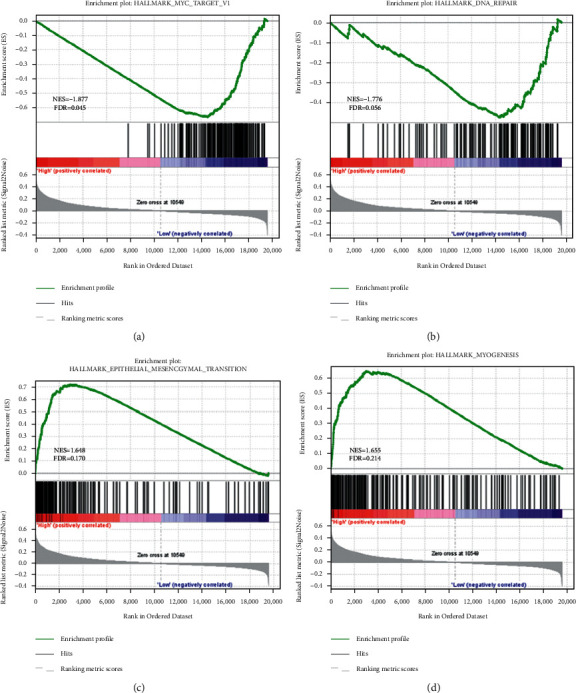
GESA results of the risk group defined by the 8-IRlncRNA prognostic signature. (a) and (b) Enrichment pathways for low-risk group. (c) and (d) Enrichment pathways for high-risk group.

We performed GSEA on the high-risk and low-risk groups to uncover the possible molecular mechanisms of 8-IRlncRNA prognostic signatures in GAD. The results show that the low-risk group was mainly enriched in myc targets and DNA repair (Figures 5(a) and 5(b)). The high-risk group was significantly enriched in the epithelial-mesenchymal transition and myogenesis pathways (Figures 5(c) and 5(d)). These pathways are all related to the occurrence and development of tumors.

### 3.6. Association of 8-IRlncRNA Signatures with Somatic Mutation Status

**Figure 6:**
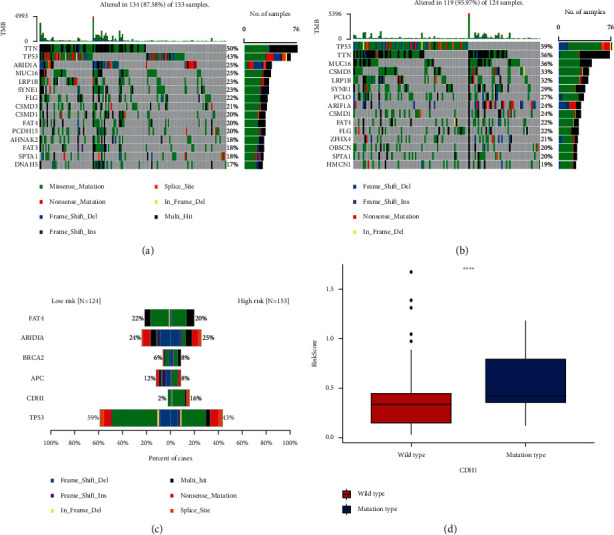
Somatic mutation status between high- and low-risk groups. (a) The top 15 gene mutations in the high-risk group. (b) The top 15 gene mutations in the low-risk group. (c) Top 5 differentially mutated genes between high-risk and low-risk groups. (d) Differential expression of CDH1 in high-risk and low-risk groups. ^∗∗∗∗^*P* < 0.0001.

The results of the analysis show that the mutation rate of genes was higher (>15%) in both the high-risk group and the low-risk group, especially in the TTN and TP53 genes (Figures 6(a) and 6(b)). The mutation rate of CDH1 (cadherin 1) was significantly higher in the high-risk group than in the low-risk group (16% vs. 2%, *P* < 0.0001) (Figures 6(c) and 6(d)). The results of our analysis are consistent with those reported in previous studies. A number of GC-related cohort studies have shown that CDH1 has a high mutation rate and thus is a high-risk factor for the high frequency of GC [23, 24].

### 3.7. Correlation between the 8-IRlncRNA Prognostic Signature and Immune Cell Infiltration

**Figure 7:**
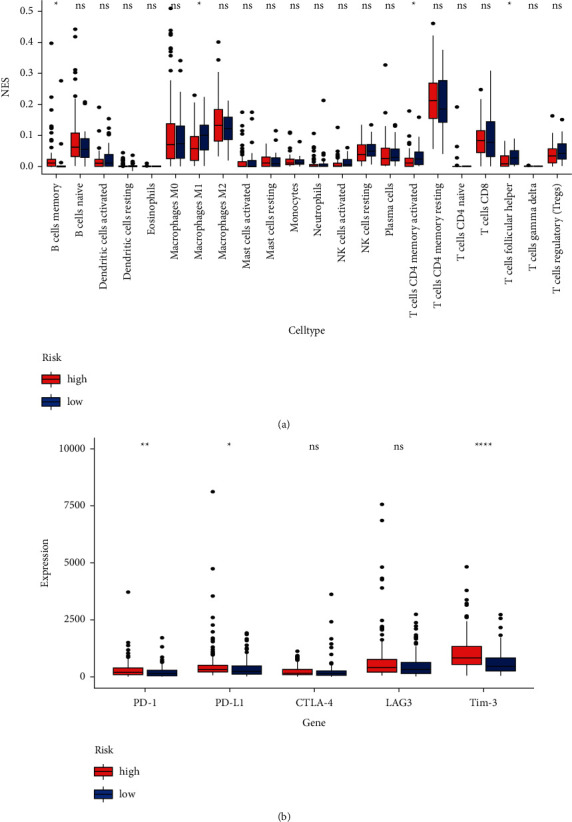
(a) The difference in 22 tumor-infiltrating immune cells between high-risk groups and low-risk groups. (b) The relationship between 8-IRlncRNA prognostic signature and response to ICI. ^∗^*P* < 0.05, ^∗∗^*P* < 0.01, ^∗∗∗∗^*P* < 0.0001.

We analyzed the abundance of 22 tumor-infiltrating immune cells in the high-risk and low-risk groups. Compared with the low-risk group, the ratio of B memory cells in the high-risk group increased, while the ratio of macrophages M1, T cells CD4 memory activated, and T follicular helper cells decreased (Figure 7(a)). These results indicate that the 8-IRlncRNA prognostic signature may be related to prognosis by affecting the immune cell infiltration in GAD.

### 3.8. Association of the Risk Signature with Immunotherapy Sensitivity

ICI, as a kind of tumor immunotherapy, is a promising treatment method for GC. Immune checkpoint molecules include PD-1, PD-L1, cytotoxic T lymphocyte-associated protein 4 (CTLA-4), T-cell immunoglobulin mucin-3 (TIM-3), and others [25]. We explored the potential of 8-IRlncRNA signatures to predict response to ICI and evaluated which risk group patients have a better response to ICI. The expression levels of PD-1, PD-L1, and TIM-3 were all upregulated, indicating that patients in the high-risk group were promising candidates for ICI (Figure 7(b)). Our results indicate that the established model has potential predictive value for immunotherapy.

## 4. Discussion

GC has the characteristics of high incidence and low 5-year survival rate [26, 27]. It is urgent to find biomarkers that can predict the progression of GAD and drug treatment sensitivity. LncRNA is involved in the proliferation, invasion, and metastasis of various tumors. In GC, knockdown of LncRNA AK023391 inhibits cells proliferation and induces apoptosis [28]. After lncRNA HOXC-AS3 was activated, it promoted the proliferation and migration of GC cells by interacting with YBX1 [29]. These reports suggest that lncRNAs can affect the occurrence and development of GC and thus affect the prognosis of cancer patients. In this study, we identified an 8-IRlncRNA prognostic signature and evaluated its prognostic value in GAD.

We identified seven IRlncRNAs (AC010890.1, AC093850.2, CH17-118O6.3, RP11-115H13.1, RP11-1260E13.4, RP11-497E19.1, and RP11-54A9.1) that were associated with high risk. Only one IRlncRNA (RP11-489D6.2) was associated with low risk. According to the risk scores generated by the expression levels of these 8 IRlncRNAs, we divided GAD patients into a low-risk and a high-risk group. Our results showed that the prognosis of the two groups were different, and the high-risk group had a poorer OS. The AUC values of 1-year, 3-year, and 5-year OS were higher in the training set, validation set, and all gastric adenocarcinoma patient cohorts. These results suggest that this 8-IRlncRNA signature has the potential to differentiate the prognosis of GAD patients. Among these 8 IRlncRNAs, knockout of AC093850.2 (LINC00460) inhibits tumorigenesis in vivo and the growth and invasion of cervical cancer cells in vitro. These studies show that AC093850.2 plays a carcinogenic role in cervical cancer [30]. Overexpression of AC093850.2 promotes the proliferation, invasion, and migration of bladder cancer cells. AC093850.2 also is related to the shorter overall survival of bladder cancer patients [31]. Our GSEA analysis showed that the 8-IRlncRNA prognostic signature was enriched in myc targets. Zhang et al. found that AC093850.2 is highly expressed in gastric cancer tissues, and its high expression is associated with lymph node metastasis, advanced TNM staging, poor disease-free survival, and poor OS. In vitro, downregulation of AC093850.2 is found to downregulate the expression of c-Myc and *β*-catenin, which suggests that AC093850.2 can promote gastric cancer cell proliferation and invasion by activating Wnt/*β*-catenin signaling [32, 33]. In in vitro experiments, RP11-54A9.1 (LINC02407) increases the malignancy of GC cells, promotes the invasion of GC cells, and decreases cell apoptosis [34]. In addition, unreported lncRNAs may be potential prognostic factors related to the occurrence and progression of GAD, but their functions remain poorly understood. Based on the GC data in the TCGA database, TCGA defines four main molecular subtypes of GC, which include MSI, EBV, GS, and CIN. These molecular subtypes can provide guidance for targeted drugs because they display unique genomic characteristics [35]. Subsequent research has shown that the EBV subtype has the best prognosis and the GS subtype has the worst prognosis. OS is lower in patients with MSI and CIN subtypes than in patients with the EBV subtype, but OS is higher than in patients with the GS subtype [36]. Our analysis shows that the GS subtype has the highest risk score, while the EBV subtype has the lowest risk score, consistent with previous studies. Mutations in the CDH1 gene are the most frequently detected germline mutations in gastric cancer and are responsible for the development of hereditary diffuse gastric cancer (HDGC) syndrome [37]. It has been reported that patients with germline mutations in the CDH1 gene have a very high cumulative lifetime risk of developing diffuse GC [38]. Patients tend to have poorer clinical outcomes when HDGC is diagnosed at a later stage. The American College of Gastroenterology recommends prophylactic gastrectomy for CDH1 mutation carriers [39]. In our study, the CDH1 gene was significantly mutated in the high-risk group. This illustrates the potential of our 8-IRlncRNA signature to predict gene mutation status in GAD and identify CDH1 gene mutations to help GAD patients decide whether to undergo surgical treatment and improve patient outcomes. These results all suggest that the 8-lncRNA signatures we identified have the potential to discriminate and predict prognosis for GAD patients.

The tumor immune microenvironment is of great value in prognostic research on GC [40]. In order to further explore the relationship between the 8-IRlncRNA signature and immune-related features, we performed immune infiltrating cell analysis on the high-risk and low-risk groups. The results show that the proportion of B memory cells increased in the high-risk group while the proportion of macrophages M1, T cells CD4 memory activated, and T cells follicular helper decreased. Tumor-infiltrating memory B cells are associated with the progression of GC [22]. Xie et al. demonstrated that inhibiting the polarization of macrophages to M1 promotes the invasion and metastasis of GC cells [41]. Follicular helper T cell-mediated suppression of IL-10^+^ B cells may increase the risk of GC development; once cancer develops, suppression of IL-10^+^ B cells may enhance overall antitumor immunity [42]. Immunotherapy has been used in the treatment of GC as an effective tumor treatment method [43]. ICI, a novel immunotherapy method, has been applied to the treatment of GC. It is well known that the expression of immune checkpoints can predict the potential of patients to benefit from ICI [44]. Immune checkpoint molecules enhance the immune response and sensitivity to ICIs by eliminating T cell activation [45]. In our study, we found that three immune checkpoints PD-1, PD-L1, and TIM-3 were significantly overexpressed in the high-risk patient group. Some studies have found that the survival time of patients with positive PD-L1 expression in gastric cancer tissue is significantly shortened [46, 47]. Two phase III clinical trials have reported that there is a positive correlation between higher PD-L1 expression in gastric cancer tissues and better treatment outcomes, but negative PD-L1 expression is a negative predictor of pembrolizumab survival benefit [21, 48]. Wang et al. also pointed out that gastric cancer patients with PD1/PD-L1 high expression may be potential beneficiaries of PD1/PD-L1 immunotherapy [49]. The upregulation of TIM-3 expression in gastric cancer is associated with poor prognosis, tumor lymph node metastasis, and advanced clinical staging of gastric cancer patients [50, 51]. Koyama et al. found that the failure of anti-PD-1 therapy was related to the upregulation of TIM-3 expression [52]. Anti-TIM-3 monoclonal antibodies can block the TIM-3 receptor to ensure immune tolerance [53]. Our results are consistent with the conclusions of existing studies, and we speculate that high-risk patients may benefit more from ICI.

This is the first study to identify an 8-IRlncRNA signature with good potency, including in predicting prognosis and response to immunotherapy, and also it is one of the few studies that combines the identification of IRlncRNA signature with somatic mutation analysis. The limitations of our study are as follows: there is lack of a suitable external dataset to validate our model, necessitating the use of data obtained from the TCGA database; and some of the lncRNAs in our identified signatures have not been characterized, and so additional in vitro and in vivo experiments are needed.

## 5. Conclusions

Gastric adenocarcinoma (GAD) is one of the most common tumors in the world and the prognosis remains very poor. It is necessary to develop biomarkers that can be used to predict the prognosis of gastric cancer. We established a prognostic signature of 8-IRlncRNA in GAD based on the LASSO model and verified that this signature can be used as a prognostic marker for GAD. We also explored the relationship between this prognostic signature and tumor immune characteristics, and the role of the 8-IRlncRNA prognostic signature in predicting immunotherapy response. However, the application value of this signature needs further validation so as to provide reliable evidence for clinical use.

## Figures and Tables

**Table 1:** Clinical pathological characteristics of included patients.

Characteristics	Training set (*N* = 195)	Validating set (*N* = 84)	Entire GC patient cohort (*N* = 279)
Age (years)			
Age <60	61 (31.3%)	30 (35.7%)	91 (32.6%)
Age ≥60	134 (68.7%)	54 (64.3%)	188 (67.4%)
Gender			
Female	71 (36.4%)	33 (39.3%)	104 (37.3%)
Male	124 (63.6%)	51 (60.7%)	175 (62.7%)
Pathologic stage			
Stage I	25 (12.8%)	14 (16.7%)	39 (14.0%)
Stage II	70 (35.9%)	27 (32.1%)	97 (34.8%)
Stage III	84 (43.1%)	34 (40.5%)	118 (42.3%)
Stage IV	16 (8.2%)	9 (10.7%)	25 (9.0%)
Inventory status			
Alive	117 (60.0%)	45 (53.6%)	162 (58.1%)
Dead	78 (40.0%)	39 (46.4%)	117 (41.9%)

**Table 2:** The details of prognostic eight immune-related lncRNAs in the training set.

Gene symbol	Ensembl ID	Coefficient
AC010890.1	ENSG00000226953	0.0082
AC093850.2	ENSG00000230838	0.0015
CH17-118O6.3	ENSG00000275585	0.0148
RP11-115H13.1	ENSG00000273906	0.0004
RP11-1260E13.4	ENSG00000262061	0.0036
RP11-489D6.2	ENSG00000259446	−0.0009
RP11-497E19.1	ENSG00000205562	0.0106
RP11-54A9.1	ENSG00000257219	0.0005

## Data Availability

The data used in this study were downloaded from The Cancer Genome Atlas (TCGA). The data of TCGA are free for people to download for scientific research.

## Supplementary Materials

Supplementary Materials Supplementary Figure 1: functional enrichment analysis of DEIRmRNA. (A) Top 10 BP terms of all DEIRmRNA; (B) top 10 CC terms of all DEIRmRNA; (C) top 10 MF terms of all DEIRmRNA; (D) top 10 KEGG pathways of all DEIRmRNA. Supplementary Figure 2: validation of 8-IRlncRNA prognostic signature in the test set. (A) Patients were divided into high- and low-risk groups based on 8-IRlncRNA in the test set. (B) The survival status of GC patients in the test set. (C) Heatmap of expression profiles of 8-IRlncRNA. (D) Survival analysis of high- and low-risk groups. (E) Time-dependent ROC curve of the 8-IRlncRNA prognostic signature. Supplementary Figure 3: validation of 8-IRlncRNA prognostic signature in the entire STAD patient cohort. (A) Patients were divided into high- and low-risk groups based on 8-IRlncRNA in the entire STAD patient cohort. (B) The survival status of GC patients in the entire STAD patient cohort. (C) Heatmap of expression profiles of 8-IRlncRNA. (D) Survival analysis of high- and low-risk groups. (E) Time-dependent ROC curve of the 8-IRlncRNA prognostic signature.
