# Supplementary figures and images for: An Immunity-Associated lncRNA Signature for Predicting Prognosis in Gastric Adenocarcinoma
Source: J Healthc Eng. 2022 Apr 25;2022:3035073. doi: 10.1155/2022/3035073 (PMC9061059; doi:10.1155/2022/3035073)

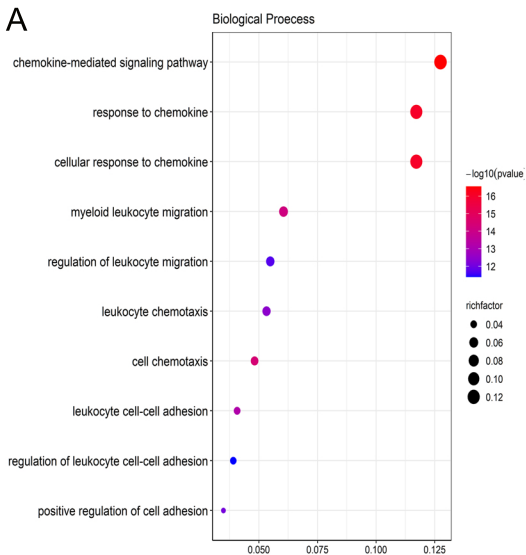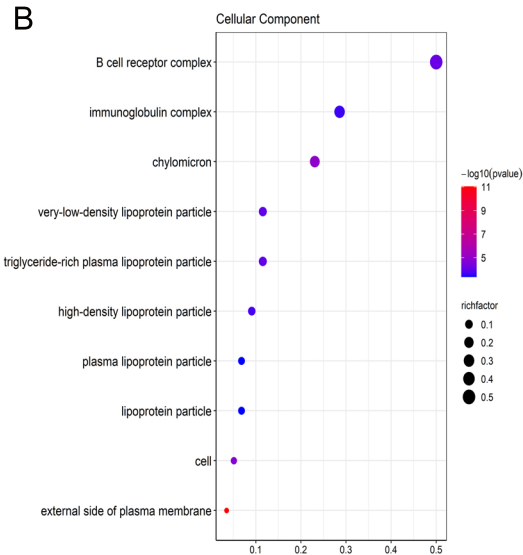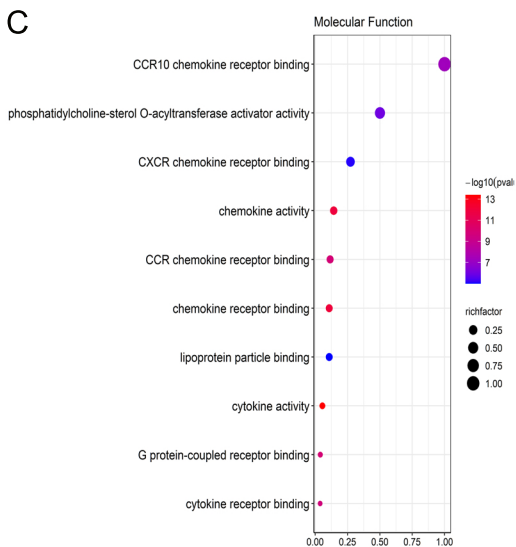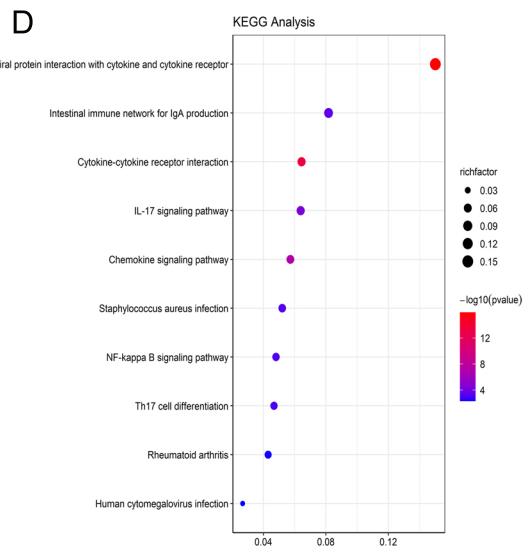

Supplement: Supplementary Materials — Supplementary Figure 1: functional enrichment analysis of DEIRmRNA. (A) Top 10 BP terms of all DEIRmRNA; (B) top 10 CC terms of all DEIRmRNA; (C) top 10 MF terms of all DEIRmRNA; (D) top 10 KEGG pathways of all DEIRmRNA. Supplementary Figure 2: validation of 8-IRlncRNA prognostic signature in the test set. (A) Patients were divided into high- and low-risk groups based on 8-IRlncRNA in the test set. (B) The survival status of GC patients in the test set. (C) Heatmap of expression profiles of 8-IRlncRNA. (D) Survival analysis of high- and low-risk groups. (E) Time-dependent ROC curve of the 8-IRlncRNA prognostic signature. Supplementary Figure 3: validation of 8-IRlncRNA prognostic signature in the entire STAD patient cohort. (A) Patients were divided into high- and low-risk groups based on 8-IRlncRNA in the entire STAD patient cohort. (B) The survival status of GC patients in the entire STAD patient cohort. (C) Heatmap of expression profiles of 8-IRlncRNA. (D) Survival analysis of high- and low-risk groups. (E) Time-dependent ROC curve of the 8-IRlncRNA prognostic signature. [file 3035073.f1.zip › 3035073.f1/Supplementary Figure 1 (1).pdf]

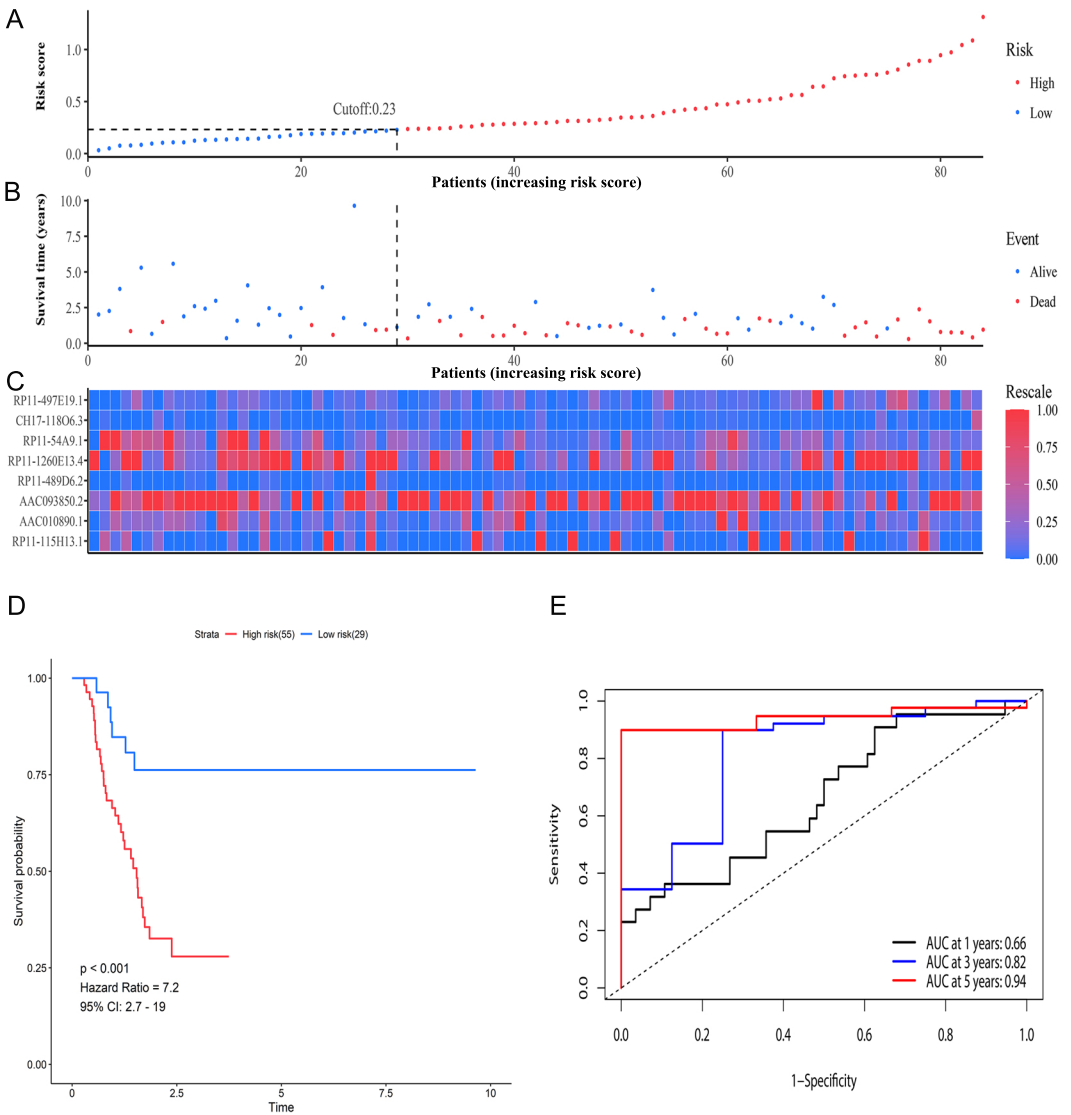

Supplement: Supplementary Materials — Supplementary Figure 1: functional enrichment analysis of DEIRmRNA. (A) Top 10 BP terms of all DEIRmRNA; (B) top 10 CC terms of all DEIRmRNA; (C) top 10 MF terms of all DEIRmRNA; (D) top 10 KEGG pathways of all DEIRmRNA. Supplementary Figure 2: validation of 8-IRlncRNA prognostic signature in the test set. (A) Patients were divided into high- and low-risk groups based on 8-IRlncRNA in the test set. (B) The survival status of GC patients in the test set. (C) Heatmap of expression profiles of 8-IRlncRNA. (D) Survival analysis of high- and low-risk groups. (E) Time-dependent ROC curve of the 8-IRlncRNA prognostic signature. Supplementary Figure 3: validation of 8-IRlncRNA prognostic signature in the entire STAD patient cohort. (A) Patients were divided into high- and low-risk groups based on 8-IRlncRNA in the entire STAD patient cohort. (B) The survival status of GC patients in the entire STAD patient cohort. (C) Heatmap of expression profiles of 8-IRlncRNA. (D) Survival analysis of high- and low-risk groups. (E) Time-dependent ROC curve of the 8-IRlncRNA prognostic signature. [file 3035073.f1.zip › 3035073.f1/Supplementary Figure 2 (1).pdf]

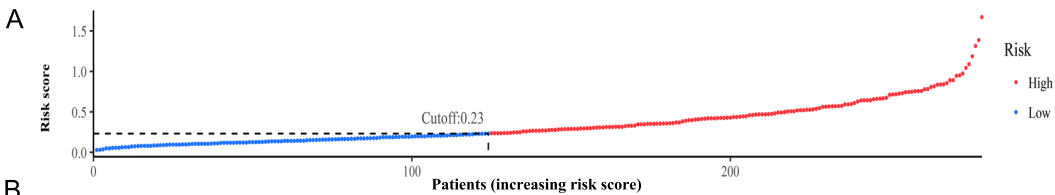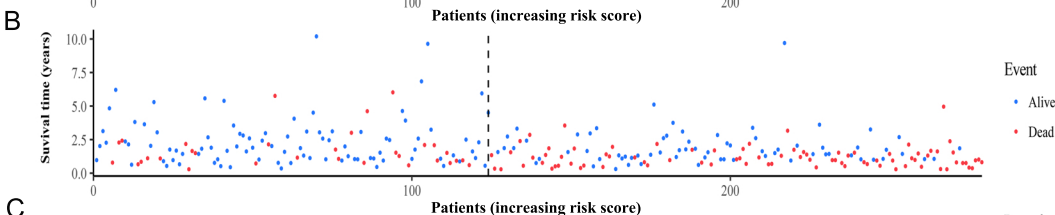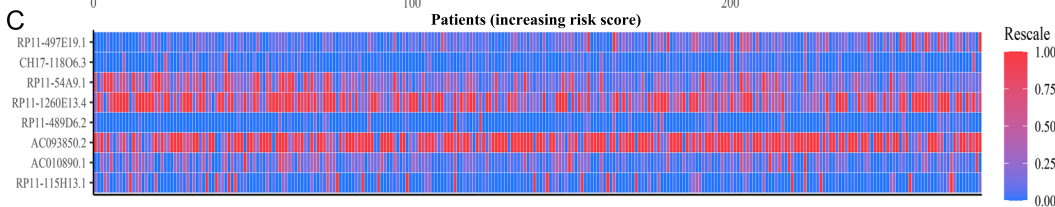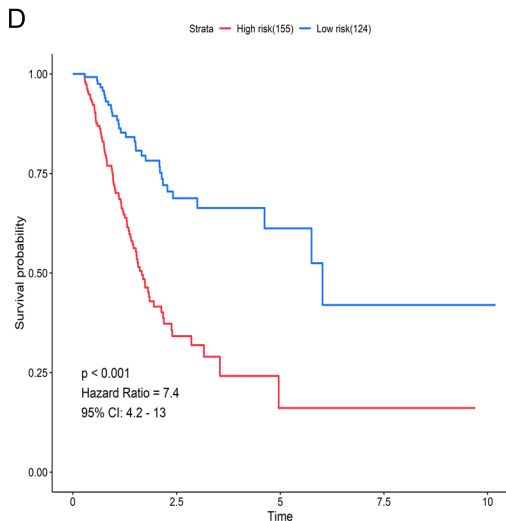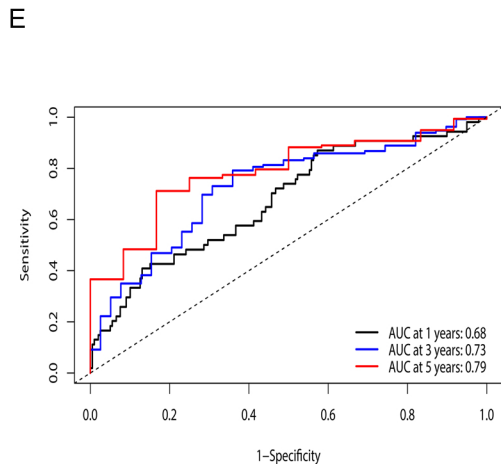

Supplement: Supplementary Materials — Supplementary Figure 1: functional enrichment analysis of DEIRmRNA. (A) Top 10 BP terms of all DEIRmRNA; (B) top 10 CC terms of all DEIRmRNA; (C) top 10 MF terms of all DEIRmRNA; (D) top 10 KEGG pathways of all DEIRmRNA. Supplementary Figure 2: validation of 8-IRlncRNA prognostic signature in the test set. (A) Patients were divided into high- and low-risk groups based on 8-IRlncRNA in the test set. (B) The survival status of GC patients in the test set. (C) Heatmap of expression profiles of 8-IRlncRNA. (D) Survival analysis of high- and low-risk groups. (E) Time-dependent ROC curve of the 8-IRlncRNA prognostic signature. Supplementary Figure 3: validation of 8-IRlncRNA prognostic signature in the entire STAD patient cohort. (A) Patients were divided into high- and low-risk groups based on 8-IRlncRNA in the entire STAD patient cohort. (B) The survival status of GC patients in the entire STAD patient cohort. (C) Heatmap of expression profiles of 8-IRlncRNA. (D) Survival analysis of high- and low-risk groups. (E) Time-dependent ROC curve of the 8-IRlncRNA prognostic signature. [file 3035073.f1.zip › 3035073.f1/Supplementary Figure 3 (1).pdf]
